# Incentives to Stimulate European Wheat Farmers to Adapt Their *Fusarium* Species Mycotoxin Management

**DOI:** 10.3390/toxins13020144

**Published:** 2021-02-14

**Authors:** Esmée M. Janssen, Monique C. M. Mourits, Alfons G. J. M. Oude Lansink, H. J. van der Fels-Klerx

**Affiliations:** Business Economics Group, Wageningen University & Research, Hollandseweg 1, 6706 KN, Wageningen, The Netherlands; esmee.m.janssen@gmail.com (E.M.J.); monique.mourits@wur.nl (M.C.M.M.); Alfons.OudeLansink@wur.nl (A.G.J.M.O.L.)

**Keywords:** mycotoxin, *Fusarium* spp., farmer, incentive, wheat, Fusarium Head Blight

## Abstract

*Fusarium* species infection in wheat can lead to Fusarium Head Blight (FHB) and contamination with mycotoxins. To fully exploit more recent insights into FHB and mycotoxin management, farmers might need to adapt their agronomic management, which can be stimulated through incentives. This study aimed to identify incentives to stimulate European farmers to adapt their agronomic management to reduce FHB and related mycotoxins in wheat. A questionnaire was distributed among 224 wheat farmers from Italy, the Netherlands, Serbia, and the United Kingdom. Using the respondents’ data, Bayesian Network modelling was applied to estimate the probability that farmers would adapt their current agronomic management under eight different incentives given the conditions set by their farm and farmer characteristics. Results show that most farmers would adapt their current agronomic management under the incentives “paid extra when wheat contains low levels of mycotoxins” and “wheat is tested for the presence of mycotoxins for free”. The most effective incentive depended on farm and farmer characteristics, such as country, crop type, size of arable land, soil type, education, and mycotoxin knowledge. Insights into the farmer characteristics related to incentives can help stakeholders in the wheat supply chain, such as farmer cooperatives and the government, to design tailor-made incentive plans.

## 1. Introduction

*Fusarium* species infection in wheat can cause Fusarium Head Blight (FHB) and contamination with mycotoxins, leading to yield losses and a decrease in food quality and safety. Occurrence studies show that *Fusarium* mycotoxins, such as zearalenones, fumonisins, and the trichothecenes deoxynivalenol (DON), nivalenol, and T-2/HT-2 toxins, are regularly found above the European Union (EU) legal limit in wheat and wheat products [1,2,3]. Mycotoxins are chemically stable substances and difficult to remove further along the wheat supply chain, implying the high relevance of prevention and control of *Fusarium* spp. infection in the field by agronomic management. Exposure assessments show that the European human intake of *Fusarium* mycotoxins is close to the tolerable daily intake for some subpopulations [2,4,5,6,7]. The occurrence of FHB and related toxins in wheat depends mainly on climate and local weather conditions [8,9,10] and farmers cope with these weather-induced risks by implementing different agronomic management measures to reduce FHB and mycotoxin contamination. One of the most efficient ways to reduce FHB and mycotoxin contamination, especially when weather or environmental conditions are favorable for fungal infection, is the implementation of an integrated agronomic approach of pre-harvest measures [11,12,13,14,15,16]. Currently, a variety of agronomic management measures is implemented by EU farmers to reduce FHB and mycotoxins [17,18,19], resulting in different management approaches among farmers. For instance, Janssen, Mourits, van der Fels-Klerx and Oude Lansink [17] showed that only 50% of Dutch farmers used an effective integrated agronomic approach consisting of a *Fusarium* spp. resistant wheat variety, the application of fungicides during flowering, and crop rotation and/or ploughing [15,20]. Hence, at farm level, there is scope for improvement of agronomic management by utilizing a more effective integrated approach. In addition to currently implemented pre-harvest measures, innovative pre-harvest measures provide additional management opportunities for wheat farmers, including novel biological control measures such as biopesticides [21,22]. Furthermore, currently applied mycotoxin management measures might not fit the envisioned changes to food production as foreseen by the European Commission’s Green Deal [23] or “sustainable agriculture” [24]. These measures propagate lower pesticide use and conservation tillage, which contrasts the effective mycotoxin reduction approach of using fungicides throughout the whole cultivation period and (deep) ploughing to burry soil debris to reduce *Fusarium* spp. infection of the next planted crop. Thus, a change in agronomic management might be needed to become more effective or to follow current technological innovations and/or political developments. 

The extent to which farmers intend to adapt their agronomic approach to manage FHB and related toxins in the coming years is unknown. Incentive mechanisms [25], such as contracts and extra payments or financial punishments, can be used to stimulate a change in farmers’ agronomic management. Information on which incentives alter a wheat farmer’s intention to adapt their agronomic management to reduce FHB and mycotoxins, in addition to insight into related farm and farmer characteristics, can be valuable when designing tailor-made incentive plans by stakeholders, such as farmer cooperatives and governmental agencies. 

This study aimed to identify which incentives stimulate different groups of European wheat farmers to adapt their agronomic management to prevent and control FHB and mycotoxin contamination in wheat.

## 2. Results

Data on intention, incentives, and characteristics of farmers were collected from 224 wheat farmers in Italy (IT), the Netherlands (NL), the United Kingdom (UK), and Serbia (RS) in 2017, using an online questionnaire. Descriptive analyses were performed to identify (i) the current intention of wheat farmers to adapt their agronomic management and (ii) farmers’ altered intention under various incentives. Subsequently, Bayesian Network (BN) modelling was used to evaluate (iii) farm and farmer characteristics related to farmers’ altered intention under various incentives, and (iv) the altered intention of specific farmer groups. More details are provided in the Methods section.

### 2.1. Farm(er) Characteristics

The questionnaire included questions on eighteen farm and farmer characteristics. Table 1 provides an overview of these variables along with the distribution of responding famers per defined category of variables. The “benchmark” variable indicates in this respect the implementation of an effective integrated agronomic approach consisting of a *Fusarium* spp. resistant wheat variety, fungicide use during flowering, and crop rotation and/or ploughing [15,20]. In the remainder of this study, this specific approach is also referred to as the “benchmark” approach.

### 2.2. Intention to Adapt the Approach

**Figure 1:**
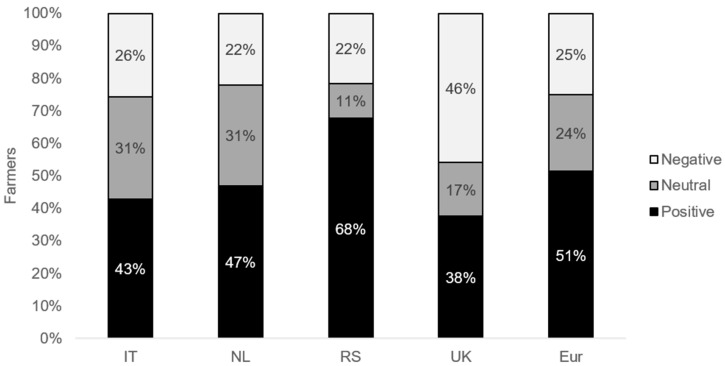
The percentage of wheat farmers from Italy (IT), the Netherlands (NL), Serbia (RS), and the United Kingdom (UK) and the combination of the four countries (Eur) with a negative, neutral, or positive intention to adapt the current agronomic management to reduce *Fusarium* spp. infection and related mycotoxin contamination.

Figure 1 shows the distribution among the responding farmers with a negative, neutral, and positive intention to adapt their *Fusarium* spp. and mycotoxin agronomic approach in the coming five years. Overall, 50% of the European wheat farmers were indicated to have a positive intention to adapt their *Fusarium* spp. and mycotoxin approach in the coming five years, ranging from 38% (UK) to 68% (RS). Twenty-five percent of the European farmers had a negative intention to adapt their approach, ranging from 22% for NL and RS to 46% for the UK. 

### 2.3. Farm and Farmer Characteristics Related to Intention (INT)

**Figure 2:**
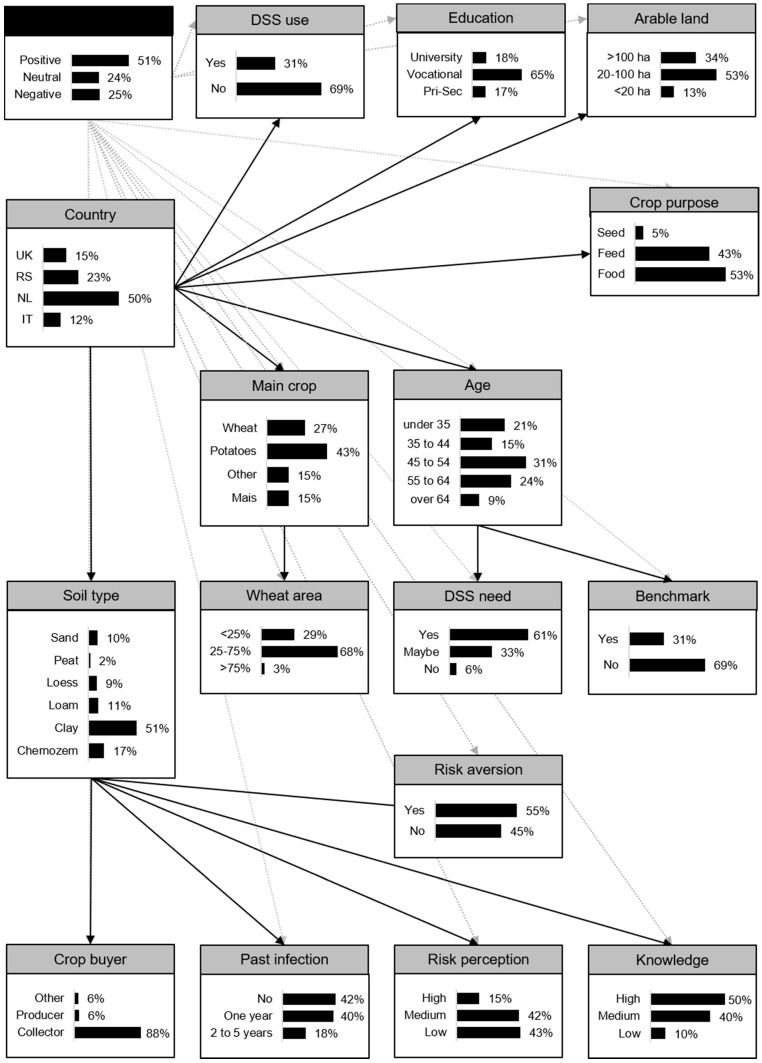
Directed acyclic graph of the Bayesian Network model for intention (INT) including the farm and farmer characteristics depicting the conditional probabilities per variable—black arrows indicate a connection between the farm and farmer characteristics, and the grey arrows indicate the connection with the conditioned variable INT. DSS = decision support system, Pri-Sec = primary or secondary education. For detailed information on the farm and farmer characteristics, see Table 1 and the Methods, Section 4.1.3.

A Bayesian Network (BN) model was developed with the basic intention to adapt the farmer’s current agronomic management to reduce *Fusarium* spp. infection and mycotoxins as the predictor value and with farm and farmer characteristics as explaining variables. BN modelling [26] is a powerful tool to explore patterns in data and to model dependencies between variables. BN models are a class of probabilistic models originating from Bayesian statistics and decision theory combined with graph theory. A BN model consists of nodes (e.g., variables such as gender) with various states (e.g., male/female) connected by arcs that reflect the dependency between the nodes. Together, these form an acyclic directed graph (DAG). A BN does not show causal relationships between nodes (variables) but statistical relations indicated by conditional probabilities. Figure 2 shows the BN DAG of the basic intention model with the probabilities per farm and farmer characteristics’ category. These results show that farmers had a probability of 51% of having a positive basic intention (INT) and 25% of having a negative basic intention to adapt their agronomic management. Farmers had a probability of 53% to “produce wheat for food” and 27% to have wheat as “main crop”. The probability that farmers had no severe “*Fusarium* spp. infection in the past” was 42%, whereas the probability that they had more than one “infection in the past” was 18% (Figure 2). The probability that the farmers used the “benchmark” approach was 31% and the probability that they received vocational “education” was 18%. 

**Figure 3:**
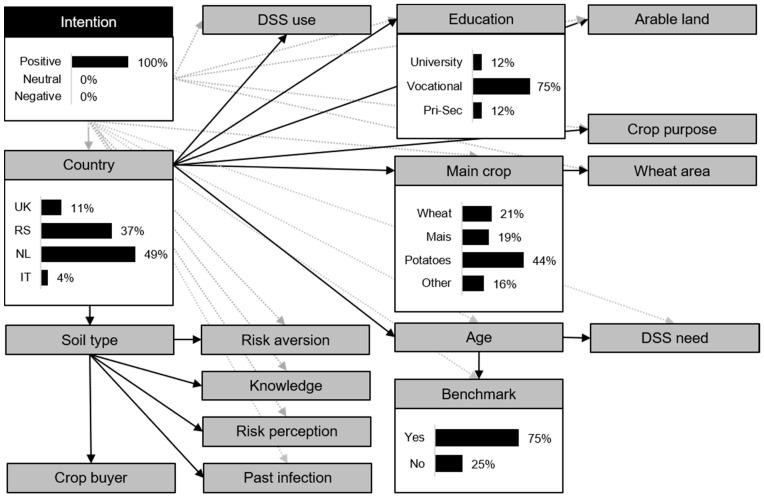
Directed acyclic graph of the Bayesian Network model for intention (INT) including the farm and farmer characteristic for farmers with a positive basic intention, depicting only the conditional probabilities of the most distinctive farm and farmer characteristics (i.e., indicating a numeric difference in conditional probabilities between farmers with a positive intention and a negative intention of >30%). The complete conditional probability table of all the farm and farm characteristics can be found in the Appendix A (Table A1). Black arrows indicate a connection between the farm and farmer characteristics and the grey arrows indicate the connection with the conditioned variable INT. DSS = decision support system; Pri-Sec = primary or secondary education. For detailed information on the farm and farmer characteristics, see Table 1 and the Methods, Section 4.1.3.

**Figure 4:**
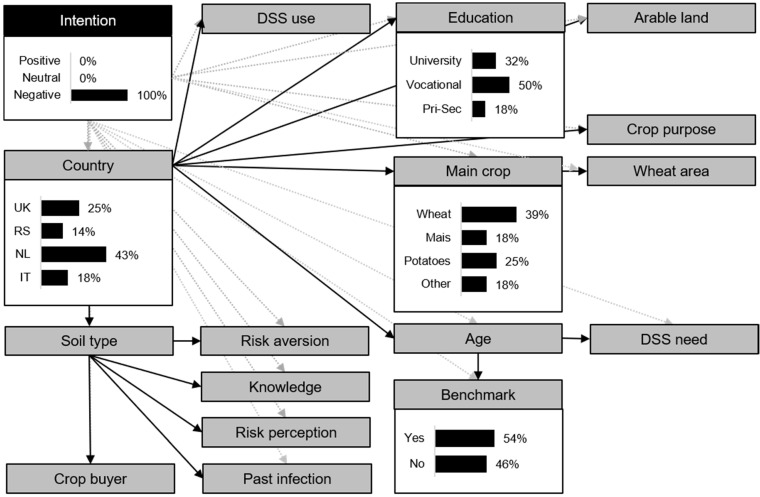
Directed acyclic graph of the Bayesian Network model for intention (INT) including the farm and farmer characteristic for farmers with a negative basic intention, depicting only the conditional probabilities of the most distinctive farm and farmer characteristics (i.e., indicating a numeric difference in conditional probabilities between farmers with a positive intention and a negative intention of >30%). The complete conditional probability table of all the farm and farm characteristics can be found in the Appendix A (Table A1). Black arrows indicate a connection between the farm and farmer characteristics and the grey arrows indicate the connection with the conditioned variable INT. DSS = decision support system, Pri-Sec = primary or secondary education. For detailed information on the farm and farmer characteristics, see Table 1 and the Methods, Section 4.1.3.

By specifying the DAG of the BN model by farmers’ basic intention category (positive versus negative), the conditional probabilities of farm and farmer characteristics of farmers with a positive basic intention (Figure 3), and of farmers with a negative basic intention (Figure 4), were indicated. For visual interpretation, Figure 3 and Figure 4 present only the conditional probabilities of the most distinctive farm and farmer characteristics (i.e., indicating a numeric difference in conditional probabilities between farmers with a positive intention and a negative intention of >30%). The complete conditional probability table of all the farm and farm characteristics can be found in the Appendix A (Table A1).

Farmers with a positive intention were most likely to come from the NL (49%) and RS (37%) and were less likely to originate from the UK (11%) or IT (4%) (Figure 3). Farmers with a negative intention were most likely to come from the NL (43%) and the UK (25%) and less likely from IT (18%) and RS (14%) (Figure 4). Wheat was more likely to be the *main crop* of farmers with a negative intention (39%), but not of farmers with a positive intention (21%). Farmers with both a positive and negative intention were most likely to use a *benchmark* approach. However, the probability levels differed, namely 75% for farmers with a positive intention and 54% for farmers with a negative intention. The probability that a farmer had obtained a university degree was 32% for farmers with a negative intention, whereas this was only 12% for farmers with a positive intention. The likelihood that farmers with a positive intention received vocational *education* was 75% and for farmers with a negative intention, this was 50%.

### 2.4. Incentivization of Farmers

The influence of eight incentive mechanisms on the basic intention of farmers to change their agronomic approach for *Fusarium* spp. and mycotoxin management was studied. Incentives included:(i) “paid extra” (getting paid extra when wheat contains low levels of mycotoxins); (ii) “paid less” (getting paid less when wheat contains too high levels of mycotoxins); (iii) “no delivery” (not being allowed to deliver the wheat after harvest because of high mycotoxin levels); (iv) “free test” (a test for mycotoxin presence in the wheat is performed for free); (v) “contract” (a multiyear contract with the buyer to deliver wheat for a fixed price); (vi) “insurance” (taking out insurance for high mycotoxin levels); (vii) “free advice” (getting free advice on agronomic management to reduce FHB), and (viii) “law” (a change in agronomic management is enforced by (inter)national law).

**Figure 5:**
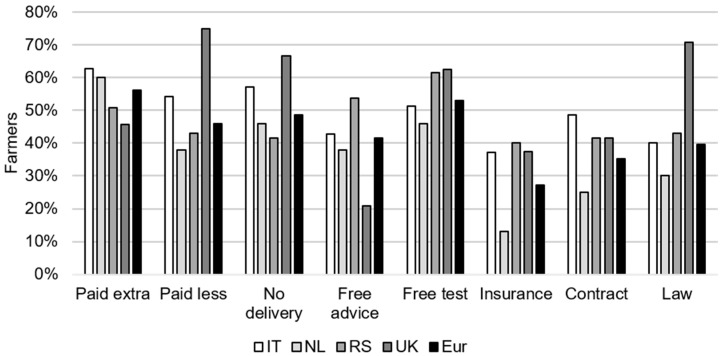
The percentage of wheat farmers from Italy (IT), the Netherlands (NL), Serbia (RS), and the United Kingdom (UK) and the combination of the four countries (Eur) who were incentivized per incentive mechanism (i.e., had an increased intention to adapt their current agronomic management to reduce *Fusarium* spp. infection and related mycotoxin contamination in comparison to the base situation without an incentive mechanism).

The percentages of farmers who were incentivized to adapt their *Fusarium* spp. and mycotoxin management approach under the evaluated incentives (reflected by an increased intention in comparison to the base situation without incentive) are depicted in Figure 5. The percentage of incentivized farmers ranged from 27% for the incentive “insurance” to 56% for the incentive “paid extra”. The two incentives that incentivized the highest percentage of farmers were “paid extra” and “free test”. These two incentives also had the least variance between the farmers among the four evaluated countries; the percentages of farmers who were incentivized ranged from 46% to 63% over the countries for both incentives. In contrast, this variation was higher among the farmers who were incentivized under “paid less”, ranging from 38% (NL) to 75% (RS), and under “law”, ranging from 30% (NL) to 71% (UK). 

Per country, the highest percentage of incentivized farmers was as follows: for IT, “paid extra” (63%) and “no delivery” (57%); for NL, “paid extra” (60%), “no delivery”(46%), and “free test” (46%); for RS, “free test” (62%) and “free advice” (54%); for the UK, “paid less” (75%) and “law” (71%).

The probabilities of each farm and farmer characteristic conditional to a decreased, increased, or unaltered intention under a particular incentive mechanism (INC) are shown in the Appendix A (Table A2). Table 2 shows a selection of the most distinctive results, indicating a large numeric difference (>30%) in the conditional probabilities of a (farm or farmer) characteristic category between farmers who were incentivized (i.e., had an increased intention) or not (i.e., had a decreased intention). The results for the incentives “paid less”, “free test”, and “law” are not shown because the differences between the conditional probabilities of the farmers with a decreased or increased intention under these incentives were small. 

Table 2 shows that farmers with an increased or unaltered intention under the incentive “paid extra” were most likely to originate from NL (56% and 54%, respectively). Farmers with a decreased intention were most likely to come from RS (33%) and the UK (33%). Farmers with an increased or unaltered intention were most likely to have clay as *soil type* (60% and 57%, respectively). Farmers with a decreased intention were most likely to have a large ‘arable’ farm (61%). Farmers with both an increased and unaltered intention were most likely to have a medium-sized farm (61% and 60%, respectively). In general, under the incentive “paid extra”, farmers were more likely to use a “benchmark” approach; however, the probability of using a benchmark approach was higher for farmers with a decreased intention (77%) and an unaltered intention (82%) than for farmers with an increased intention (52%). Farmers with a decreased intention were most likely to be “risk-averse” (72%). This probability was lower for farmers with an unaltered intention (61%) or an increased intention (48%). 

Under the incentive “no delivery”, all farmers (so independent of the INC state of increased, decreased, or unaltered intention) were most likely to have received vocational “education”. However, farmers with increased intention were also likely to have received university “education” (31%), whereas farmers with a decreased intention were not likely to have received university “education” (0%) (Table 2). 

Farmers with a decreased intention under the incentive “free test” were most likely to have potatoes as the *main crop* (68%) and not maize (0%). For farmers with an increased intention under the incentive “free test”, the *main crop* was most likely to be wheat (35%) or potatoes (32%), while the likelihood to have maize as the *main crop* was 16%. All farmers under the incentive “free test” were most likely to have received vocational *education*; the probabilities were 89% for farmers with a decreased intention, 53% for farmers with an increased intention, and 72% for farmers with an unaltered intention. Farmers with a decreased intention under incentive “free test” were most likely to originate from NL (73%).

Farmers with a decreased intention under the incentive “insurance” were most likely to have potatoes as the “main crop” (59%). For farmers with an unaltered intention, this was 39%, while for farmers with an increased intention, this was 11%. Farmers with an increased intention had either wheat (44%) or maize (22%) as their “main crop”. The farmers with a decreased or unaltered intention under the incentive “insurance” were most likely to have received vocational “education” (84% and 57%, respectively). The likelihood for farmers with an increased intention was the same for each “education” category (33% for each of primary/secondary school; vocational; university). Farmers with an increased intention under the incentive “insurance” were most likely to have a high mycotoxin “knowledge” level (74%). In contrast, for farmers with a decreased intention, this probability was 40%; these farmers were more likely to have a medium “knowledge” score (54%). Farmers with a decreased intention under incentive “insurance” were most likely to come from NL (66%), whereas farmers with an increased intention were most likely to come from RS (30%) or IT (26%). 

Farmers with a decreased intention under the incentive “contract” were most likely to have potatoes as the “main crop” (60%) and to “produce” wheat for feed (63%), whereas for farmers with an increased intention, the “main crop” was most likely to be wheat (41%) produced for food (64%). 

### 2.5. Targeting Specific Farmer Groups

**Figure 6:**
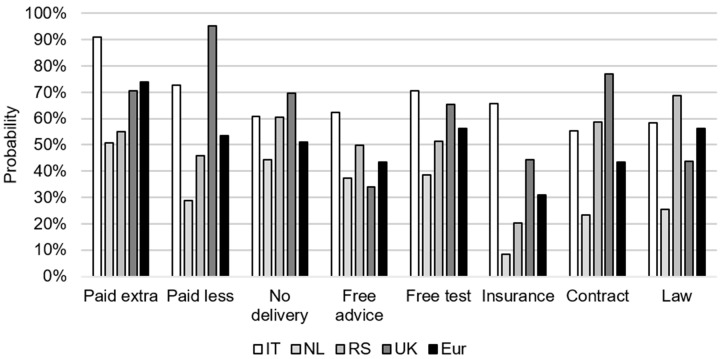
The probability of wheat farmers from Italy (IT), the Netherlands (NL), Serbia (RS), and the United Kingdom (UK) and the combination of the four countries (Eur) who did not use the “benchmark” approach (scenario 1) with an increased intention under each of the eight incentives.

Scenario analyses were performed by specifying certain farm and farmer characteristics in the INC BN models. Two scenarios were analyzed, focusing on farmers not applying the “benchmark” approach (scenario 1) and on farmers with a decreased intention to alter their management under the most promising incentive (scenario 2). The first BN scenario results are presented in Figure 6, showing that a European farmer who did not use a “benchmark” approach had a probability of 74% to be incentivized under the incentive “paid extra” and a probability of 56% to be incentivized under each of the incentives “free test” and “law”. Italian farmers who did not use the “benchmark” approach had a 91% probability to be incentivized under the incentive “paid extra” and a 73% probability under the incentive “paid less”. The highest probability for NL farmers to be incentivized was 51% under “paid extra” and 44% under “no delivery”. For RS farmers, the highest probability to be incentivized was under the incentive “law” (69%) and “no delivery” (61%). For UK farmers, the highest probability was under the incentives “paid less” (95%) and “contract” (77%).

**Figure 7:**
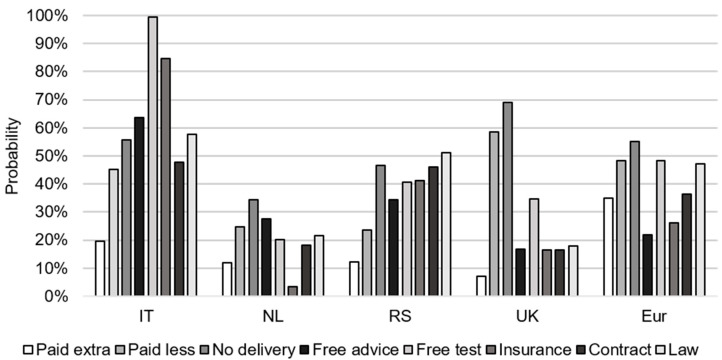
The probability of wheat farmers from Italy (IT), the Netherlands (NL), Serbia (RS), the United Kingdom (UK), and the combination of the four countries (Eur) characterized by the three most discriminating characteristics related to a decreased intention under “paid extra” (scenario 2), resulting in an increased intention under the eight incentives.

In the second BN scenario analysis, alternative incentives were identified for those farmers who had a decreased intention under the overall most promising incentive. The most promising incentive based on the largest percentage of farmers with an increased intention under this incentive was “paid extra” (Figure 5) Based on the differences in the conditional probabilities of increased and decreased farmers under “paid extra”, the three most discriminating characteristics were selected per country to define farmers with a decreased intention. The main characteristics of Italian farmers with a decreased intention under the incentive “paid extra” were no past “*Fusarium* spp. infection” (78%), growing wheat as the “main crop” (99%), and having a large “wheat area” (49%) (Table A3). When these three characteristics were submitted to the BN model of “paid extra”, results indicated that IT farmers who met these characteristics still had a 20% probability of having an increased intention (Figure 7) and an 80% probability of having a decreased intention, indicating that the selection of specific characteristics did not cover the whole group of Italian farmers with a decreased intention under “paid extra”. NL farmers with a decreased intention were characterized by having a large size of “arable land” (99%), being “risk-averse” (90%), and being in the *age* group 45–54 (49%) (Table A3). This group of farmers still had a probability of 12% to have an increased intention under the incentive “paid extra” (Figure 7) and of 45% to have a decreased intention. For RS farmers, these characteristics were having a medium “wheat area” (66%), received vocational “education” (66%), and having a low “risk perception” (67%) (Table A3), resulting in a probability of 12% to have an increased intention under the incentive “paid extra” (Figure 7) and of 60% to have a decreased intention. For UK farmers, the three main farm characteristics related to a decreased intention under incentive “paid extra” were wheat production for food “purpose” (99%), using the “benchmark” approach (81%), and being in the “age” group 55–64 (66%) (Table A3), resulting in a probability of 7% to have an increased intention (Figure 7) and of 69% to have a decreased intention under the incentive “paid extra”. The three main characteristics distinguishing European (Eur) farmers with a decreased intention from incentivized farmers under the incentive “paid extra” were related to large “arable land” (61%), the use of the “benchmark” approach (77%), and being “risk-averse” (72%) (Table A3). This group of farmers had a 35% probability to have an increased intention and 36% probability to have a decreased intention (Figure 7) under the incentive “paid extra”.

When the above-selected main farm and farmer characteristics were used to run the INC BN models, results of these scenario analyses showed that typical Italian farmers with a decreased intention under the incentive “paid extra” had the highest probability of being incentivized under the alternative incentives “free test” (99%) and “insurance” (85%) (Figure 7). The best alternative incentives to incentivize NL farmers with a decreased intention under “paid extra” were “no delivery” (34%) and “free advice” (28%). Corresponding alternative incentives for RS farmers were “law” (51%) and “no delivery” (47%), and for the UK farmers, “no delivery” (69%) and “paid less” (59%). For European farmers, the best alternative incentives were “no delivery” (55%), “free test” (48%), and “paid less” (48%) (Figure 7).

## 3. Discussion

This study analyzed the effect of several incentives on the change in farmers’ intention (incentivization). The insights of this study can be generalized to actual behavior, since a stronger intention implies that it is more likely that the behavior will be executed in the future [27]. The incentive under which the largest percentage of farmers increased their intention to adapt their FHB and mycotoxin management approach were payments when wheat contains low mycotoxin levels (“paid extra”) and testing wheat for presence of mycotoxins for free (“free test”). The exact monetary value needed to incentivize farmers with the incentive “paid extra” was not studied. Implementing the incentive “paid extra” requires the testing of mycotoxin concentrations in wheat. This testing is paired with extra costs for either the farmer or the stakeholder implementing the incentive [28]. A change in management can be paired with higher costs for the farmer and, therefore, the risk premium related to “paid extra” should be sufficient so farmers will actually change their management under this incentive. The reader is referred to Dahl and Wilson [29], who analyzed the risk and determined risk premiums necessary to induce farmers to adopt technologies to reduce FHB in wheat. Although with “paid extra”, more farmers can be incentivized to change their management approach compared to the other incentives, it might not be the preferred option for stakeholders, because of budgetary limits. In addition, although farmers indicated a preference for “paid extra”, the incentivization effect of monetary and in-kind incentives can be similar when evaluated over a longer time span [30]. This gives prospect to the in-kind incentives evaluated in the current study as well, which can be potentially studied in the longer term in a future study.

Although the incentive “paid extra” seems to be most promising, not all farmers had an increased intention under this incentive. A scenario analysis with the BN model provided insight into alternative incentives for this group of farmers. The best alternative incentive to “paid extra” differed per country, i.e., Italian wheat farmers were incentivized by multiple incentives including “free test” (99%) and “insurance” (85%), the UK farmers by “no delivery” (69%), and the Serbian farmers by “no delivery” (55%). For the Dutch farmers, the highest likelihood for an alternative incentive was only 35% for the incentive “no delivery”. This implies that Dutch wheat farmers are mainly incentivized by paying them extra when the wheat contains low mycotoxin levels, and that they were only somewhat incentivized by the other seven incentives investigated in this study. The type-casted European farmer did not fully reflect the group of farmers that was disincentivized by “paid extra”, because these typical farmers still had a probability of 35% to have an increased intention under the incentive “paid extra”. Therefore, the effect of the alternative incentives for European farmers was limited, showing only a slight increase in probability of having an increased intention under the alternative incentive “no delivery” (55%). Overall, the BN model showed alternative incentives to “paid extra”, including in-kind incentives such as “no delivery”, “free test”, and “insurance”. 

The results of this study show country differences between the “best” incentives, the “best” alternative incentives, and between specific groups of farmers. The observed differences in incentives between the countries might be related to the differences in wheat production systems, cultural differences, and/or differences in the wheat value chain and relationships among the actors in the chain. Baur, et al. [31] found differences between countries in North-West Europe regarding their openness to change, i.e., farmers in the Netherlands, Denmark, and Switzerland were less conservative and more open to change than farmers from Austria, Finland, and Germany. Country differences were also found by Fischer (2009) [32], who indicated that the prioritized choice of contract type may be highly chain- and country-specific; for example, within countries, differences between the cereal, beef, and pig meat chain were found. In this study, the likelihood that a farmer was incentivized by the incentive “contract” was, in general, low, ranging from 25% for NL farmers to 49% for IT farmers. The responses among countries may originate from the types of contracts with which the farmers are familiar. In the UK, 53% of the cereal farmers had a written contract or cross-shareholding arrangements between the farmer and processor [32]. Solazzo, et al. [33] found that only 12% of the Italian durum wheat farmers signed a forwarding contract because they lacked trust in contracts and did not want to have constraints. Moreover, they reported that turnover and degree of specialization in durum wheat production drove the adoption of written contracts. This is in line with the results of the current study, showing that farmers who were incentivized by the incentive “contract” were most likely to have wheat as main crop and produce wheat for food. These examples demonstrate the need and opportunities to design tailor-made incentive plans. 

This study applied BN modelling to identify the characteristics of farmers and their intention to adapt their agronomic management for reduction of FHB and mycotoxins. One of the strengths of BN modelling is that it can easily consider possible relationships among explanatory variables and can handle variables with a skewed distribution. For example, in this study, farms in the Netherlands and Serbia were over-represented in the study sample compared to farms in the United Kingdom and Italy; hence, the distribution of the variable “country” was skewed but could nevertheless be used in the analysis. Another strength of BN modelling is that it is possible to simulate different scenarios by selecting only a few or even many variable states and determine the probability of other variables, as is shown for farmers without the benchmark approach (scenario analysis 1). Validation of the BN models was considered acceptable: the percentage of correctly predicted responses ranged from 85% to 94% for the training set and 38% to 67% for the test set. 

This study shows that BN models can be used to select groups of farmers that need to be incentivized to change, such as farmers not applying the benchmark approach. The best (or second best) incentives can be selected for these groups, given their specific farm and farmer characteristics. Moreover, the BN model can give insight into farmer groups with specific characteristics related to an incentive selected by stakeholders. The results of this study provide a starting point for stakeholders to select potential incentives that can stimulate a change in farmers’ agronomic management to reduce FHB and mycotoxin contamination. 

The eight incentives used in this study were described in general terms and do not include any specific discrimination within the incentives, e.g., farmers’ intention under different type of contracts. There is extensive literature on the differences in, e.g., contracts [34,35], insurance [36], and premiums [29,37] in wheat and crop production. The inclusion of specific incentive mechanisms was beyond the scope of this study, but the results of this study provide interesting leads for further, more in-depth investigation—for example, to study the exact premium of the most promising incentive “paid extra” or the specific type of contract that is needed to incentivize farmers.

In conclusion, this study shows that, on average, 51% of the studied European wheat farmers had the intention to change their agronomic approach to reduce *Fusarium* spp. infection and related mycotoxin contamination. This percentage varied between the four EU countries, ranging from 38% to 67%. The most effective incentive to increase farmers’ intention to adapt their management depended on farm and farmer characteristics, such as crop type, size of arable land, soil type, education, and mycotoxin knowledge, and varied among countries. Most farmers from Italy and the Netherlands were incentivized by “paid extra” and “no delivery”; farmers from Serbia by “free test” and “free advice”, and those from the United Kingdom by “paid less” and “law”. Insights into the farmer characteristics related to incentives can help stakeholders in the wheat supply chain, such as farmer cooperatives and the government, to design tailor-made incentive plans. 

## 4. Materials and Methods

Incentives to stimulate farmers to change their agronomic management, as well as farm and farmer characteristics, were selected based on the results of a literature study and expert consultation. Questionnaires were designed to identify which incentives influence a farmer’s intention to adapt their current agronomic management. This influence was estimated by an alteration in their intention when no incentives were involved compared to when they were influenced by a certain incentive, i.e., this can be an increased (incentivized), decreased, or unaltered intention. The questionnaires were distributed among European wheat farmers from four European countries: Italy (IT), the Netherlands (NL), Serbia (RS), and the United Kingdom (UK). The questionnaire data were analyzed using descriptive analyses and Bayesian Network (BN) modelling. 

### 4.1. Selection of Variables

#### 4.1.1. Intention

According to the Theory of Planned Behavior, intentions are a proximal measure of future behavior, and the stronger the intention, the more likely the behavior will be executed in the future [27]. The main interest of this study was the current intention of farmers to adapt their agronomic approach to reduce *Fusarium* spp. infection in the coming five years, and how this intention was altered under certain incentives. An adaptation in agronomic management can entail taking fewer, more, or different pre-harvest management measures.

#### 4.1.2. Incentives

Incentive mechanisms can be used by stakeholders in the chain to enforce farmers to change their agronomic management. Lefebvre, et al. [38] describe three classes of incentives used in crop protection, namely regulatory instruments, information dissemination measures, and incentive-based instruments. Incentive-based instruments can be classified as rewards and punishments [38], and as monetary and in-kind incentives [30]. In this study, eight incentives relevant to FHB and mycotoxin management in wheat were selected, covering a range of different types of incentive mechanisms as described above. In this study, two monetary incentives—with either a reward or punishment, such as premiums and discounts—were evaluated. These were (i) “paid extra” (getting paid extra when wheat contains low levels of mycotoxins) and (ii) “paid less” (getting paid less when wheat contains too high levels of mycotoxins). Three other incentives offer an in-kind punishment, namely (iii) “no delivery” (not being allowed to deliver the wheat after harvest because of high mycotoxin levels) or an in-kind reward, namely (iv) “free test” (a test for mycotoxin presence in the wheat is performed for free) and (v) “contract” (a multiyear contract with the buyer to deliver wheat for a fixed price). Since weather is a major influential factor on *Fusarium* spp. infection and mycotoxin production, the incentive (vi) “insurance” (taking out insurance for high mycotoxin levels) was also included. The information dissemination measures were covered by the incentive (vii) “free advice” (getting free advice on agronomic management to reduce FHB) and the regulatory instruments by the incentive (viii) “law” (a change in agronomic management is enforced by (inter)national law). 

#### 4.1.3. Farm and Farmer Characteristics 

This section describes the selection of farm and farmer characteristics that are potentially related to farmers’ altered intention under various incentives and the altered intention of specific farmer groups, i.e., groups with certain farm and/or farmer characteristics. The literature suggests a range of farm and farmer characteristics that are related to agronomic management, a change in management, and incentives [17,39,40,41]. Based on the questionnaire used by Janssen, Mourits, van der Fels-Klerx and Oude Lansink [17], eighteen farm and farmer characteristics were selected for this study, of which twelve were related to the farm and six to the farmer. The twelve farm characteristics were country; organic production; arable land size [42,43,44]; the percentage of wheat production area; soil type [45,46]; main crop; purpose of wheat production (food, feed, or seed); type of buyer of the wheat; implementation of an effective integrated agronomic approach consisting of a *Fusarium* spp. resistant wheat variety, using fungicides during flowering, and crop rotation and/or ploughing [15,20], referred to as the “benchmark” approach; experience with past *Fusarium* spp. infections [47]; the use of a decision support system for FHB and mycotoxin management [48,49]; and need of a decision support system for FHB and mycotoxin management. The six farmer characteristics were age [43,50,51]; gender [52]; education level [31,51,53]; risk perception (a combination of the expected severity of an infection and its probability of occurrence [54]); risk aversion (i.e., if the farmer takes less risk than his/her peer farmers [55]); and level of knowledge [56] of FHB and mycotoxins.

### 4.2. Questionnaire

Data on intention, incentives, and characteristics of farmers were collected from wheat farmers in Italy, the Netherlands, the United Kingdom, and Serbia using an online questionnaire. The specific question and answer formats of the variables can be found in Table 1, Table 3 and Table 4. The questions were part of a questionnaire among European wheat farmers which collected information on aspects such as farm and farmer characteristics, pre-harvest measures implemented by the farmer [17], perceived (cost-)effectiveness of pre-harvest measures, and intention, with underlying behavioral constructs based on the Theory of Planned Behavior of farmers to adapt their agronomic management approach [57]. The questionnaire was designed and conducted within the European Union’s Horizon 2020 MyToolbox project [58]. The questionnaire was developed in Dutch and translated into the respective languages by native speakers. Before implementation, the questionnaire was pre-tested by three Dutch farmers for clarity and consistency. Their feedback was used to improve the questionnaire. The link to the online questionnaire was distributed via farmers’ associations in the four respective countries by email and via online newsletters. All personal information was stored separately from the questionnaire output. The study protocol and consent procedure complied with the Netherlands Code of Conduct for Scientific Practice and was approved by the Social Sciences Ethics Committee of the Wageningen University (CoC number 09131098).

### 4.3. Bayesian Network Model

With a scenario analysis, states of one or multiple nodes (e.g., a certain farmer characteristic) can be submitted to the BN model to return the conditional probability tables of the remaining variables (nodes). In this study, using the collected questionnaire data, nine BN models were fitted: one reflecting the relationships between the evaluated variables and the basic intention (INT) and one model for each of the eight incentives to reflect the relationships between the evaluated variables and the altered intention given a specific incentive (INC). The nodes represent the farm and farmer characteristics, such as “age” and “arable land”, with different states (categories such as “small” and “large”).

### 4.4. Data Processing

Data on the selected variables derived from the questionnaire were processed to be used for further analyses. A total of 332 farmers participated in the study; however, not all farmers completed the questionnaire, resulting in missing data. The responses of farmers that did not answer the questions on the variables intention (Table 3) and incentives (Table 4) were removed, resulting in a dataset containing 224 respondents. Of these responses, 35 respondents were from IT, 100 from the NL, 65 from RS, and 24 from the UK. The variables “organic production” and “gender” were removed from the dataset because of insufficient variation among the respondents for these characteristics (e.g., 98% of the farmers were male). Of the 224 respondents, 140 (63%) records were complete; the remaining respondents were missing data on maximum seven out of the eighteen variables. The variables that missed records were “education” (17%) and “age” (17%), which were questions at the end of the questionnaire, as well as “wheat area” (16%) and “arable land” (15%), which were open-ended questions. The variables that were missing in 1–5% of the records were “main crop”; “soil type”; “knowledge”; “past infection”; and “crop purpose”.

Numeric data were processed into categories, and categorical data were collapsed to reduce the number of categories per variable (Table 1), so that a discrete BN model could be applied. The variable farmer’s intention (INT) was constructed from respondents’ data for three related questions [59,60], each measured on a bipolar, textual, 5-point Likert scale (Table 3). For the analysis, this scale was converted into a numerical score ranging from −2 to 2. The answer scores (based on three questions) were measured by Cronbach alpha (Cronbach, 1951) to confirm that they were internally consistent (Cα > 0.7) and then combined into a single composite score (INT) by averaging the three scores. Each primary incentive (INCp) was directly based on a single question on a bipolar, textual, 5-point Likert scale in the questionnaire (Table 4). This score was also converted to a numerical score ranging from −2 to 2 in the analysis.

A new variable, “INC”, was created based on the primary incentive score in the questionnaire (INCp) and the basic intention score (INT), to indicate a change (increased, decreased, or unaltered) in intention in behavior under each of the eight incentives included in the study. The INC variable state was labelled “Decreased” when the score of INCp was at least 0.5 point lower than the INT score (INCp–INT ≤ −0.5), “Increased” when the score of INCp was at least 0.5 point higher than the INT score (INCp–INT≥ 0.5), and “Unaltered” when the differences in scores were less than 0.5 point (INCp-INT between [−0.5, 0.5]). Each INC variable was used for descriptive statistics and as predictor variable in the INC BN models.

The numeric INT and INCp scores were renamed: a score below zero was labelled “Negative”, a score equal to zero was “Neutral”, and a score above zero was “Positive” and included as a variable in the INC BN models. Per country, the percentage of farmers with a positive, neutral, and negative INT, and the percentage of farmers with an increased intention under the eight incentives (INCs), were calculated.

The respondents’ answers on the farm and farmer characteristic questions were categorized. The classification of the farm and farmer characteristics can be found in Table 1 (see also Janssen, Mourits, van der Fels-Klerx and Oude Lansink [17]). “Arable land” was indicated in hectares (ha) and divided into three categories: small (<20 ha), medium (20–100 ha), and large (>100 ha) [61]. The variable “wheat area” was created by dividing the continuous variable “ha wheat field” by the continuous variable “ha arable land”. The “wheat area” was then categorized into small (<25%), medium (25–75%), and large (>75%). Farmers’ “main crops” were divided into four categories: wheat, potatoes, maize, and “other crops”. The effective integrated agronomic approach consisting of a combination of a *Fusarium* spp. resistant crop, using fungicides during flowering, and crop rotation and/or ploughing [15,20], named the “benchmark” approach variable, was labelled “yes” when farmers applied the “benchmark” approach, and “no” otherwise. Five “age” classes were made: farmers under the age of 35 years were merged into the first class, while those with an age above 65 years into the fifth class. The remaining three classes were defined by 10-year increments between the age of 35 and 65 years. The classification of “education” varied greatly among countries. In the questionnaire, local names of education were used which were not always directly comparable with one another. Therefore, three broad classes of “education” were created. The first category, “pri-sec”, consisted of primary and secondary education, regardless of the level. The second category, “uni”, included university degrees such as bachelor and master studies. The remaining educational levels were classified into “vocational” education. This included, for example, vocational training and trade school. With the variable “risk aversion”, farmers who answered that they were willing to take less risk than other farmers in their community were classified as risk averse (category “yes”), the remaining farmers were classified within the category “no”. Risk perception is defined as a combination of the expected severity of an infection and its probability of occurrence [54]. The “risk perception” score was obtained by the multiplication of the scores to sub-questions on susceptibility and severity of infection (1–25), and divided into low (<7), medium (7 - 14), and high (>14) risk perception. The “knowledge” score was calculated by the sum of the scores for five knowledge statements scored as 0 (does not know or answered incorrectly) or as 1 (answered correctly) and divided into low (<2), medium (2 - 3), and high (>3) knowledge. The classification of the other variables was straightforward, as presented in Table 1.

### 4.5. BN Model Development

Constructing a BN consists of two steps: (step 1) learning the network structure (i.e., the dependency among the variables of the network) and (step 2) learning the parameters (i.e., quantitative stage that determines the conditional probabilities of each variable, given its parents) [62]. In addition, the models were validated. Hence, three different sub-datasets were created for each of the nine developed BN models: one for learning the network structure (training set 1), one for parameter learning (training set 2), and one for model validation (test set). The BN models were constructed and analyzed in the software R [63] with packages bnlearn [64] and gRain [65]. To create training set 1, first, incomplete records of all 224 records from the entire questionnaire dataset were imputed with the Expectation-Maximization (EM) algorithm [66] by the structural.em function of the R-package bnlearn [64]. Subsequently, 80% of this dataset was selected to create training set 1 (*n*= 179 records). To create training set 2, the same records were selected as for training set 1 but incomplete records were removed (training set 2; n = 112 records). The remaining 20% records of the original dataset was used for model validation (test set; n = 45 records). For each of the nine BN models (one INT and eight INCs), the variables needed were selected from training set 1, training set 2, and the test set. The INT datasets consisted of INT as dependent/predictor variable and sixteen farm and farmer characteristics variables. The INC datasets consisted of the specific INC variable (indicating the increased, decreased, or unaltered intention) as dependent/predictor variable, the sixteen farm and farmer characteristics, the basic INT and the primary INC variable (INCp). Thus, in total, nine BN models, one for each predictor variable (one INT and eight INCs), were constructed. The BN models were fitted with the Tree Augmented Naïve (TAN) Bayes algorithm. In line with the predictive modelling procedure, validation was performed with the test dataset, which was not used for constructing or training the BN [67]. In addition, an internal validation was performed with the dataset for parameter learning (training set 2). With these two validation datasets, the dependent variable was predicted (depending on the model, either INT or one of the INCs). A correct prediction was assumed when the predicted state/category with the highest probability was the same as the validation variable state/category. The percentages of correctly predicted dependent variables were calculated to present the model validations. Validation results show that the percentage of correctly predicted responses ranged from 85% to 97% for training set 2 and from 44% to 62% for the test set.

### 4.6. Farm and Farmer Characteristics Related to Intentions

With the developed BN models in place, further analyses were performed by assessing the probability tables of the BN models and the conditional probability tables of different states (characteristic) of a node (variable).

For the INT BN model, the basic probability tables for the whole model are shown (Figure 2). In addition, the conditional probabilities of the farm and farmers’ characteristics for farmers with a negative intention and with a positive intention are shown (Appendix A Table A1). For this purpose, the probability tables were depicted within the acyclic directed graphs (DAG) of the INT BN model (Figure 2, Figure 3 and Figure 4).

For each of the eight INC models, the conditional probability tables of the farm and farmer characteristics for farmers with a decreased, increased, or unaltered intention under each incentive (INC) were calculated. In addition, scenario analyses were performed by specifying certain farm and farmer characteristics in the INC BN models and returning the related conditional probabilities of the INC variables. Two scenarios were analyzed, focusing on farmers not applying the benchmark approach (scenario 1) and on farmers with a decreased intention to alter their management under the most promising incentive (scenario 2).

#### 4.6.1. Scenario 1

The benchmark approach is considered an effective approach to reduce FHB and mycotoxins in wheat, but this approach is not implemented by all farmers. Therefore, in the first scenario, promising incentives for the group of farmers currently not using the benchmark approach were identified. The BN models were used to estimate the probability of an increased intention of this group of farmers under each of the eight incentives, per country.

#### 4.6.2. Scenario 2

Not all farmers will be incentivized by the same incentives and may even have a decreased intention under certain incentives. In scenario analysis 2, alternative incentives for this group of disincentivized farmers were identified. As a case study, the focus was on the farmers who were disincentivized (i.e., having a decreased intention) by the overall most promising incentive. Therefore, first, the most promising incentive out of the eight evaluated incentives was selected based on the highest percentage of farmers who had an increased intention under this incentive. Second, based on the conditional probability tables of farmers with a decreased intention under this most promising incentive, three farm and farmer characteristics were selected. This selection of the three most discriminating characteristics was made by comparing the conditional probabilities of farmers with a decreased and increased intention under the most promising incentive, per country. Third, per country, these three selected characteristics were subsequently used to define the conditions for a scenario analysis for the BN models of each of the seven alternative incentives and the BN model for the most promising incentive for comparison. Then, this scenario analysis was run for each of the eight BN models per country (IT, NL, RS, and UK) and for Europe in general. The output shows the probability of an increased intention under the eight incentives for each of the characterized group of farmers per country.

## Figures and Tables

**Table 1:** Variable related question and answer format of the questionnaire on farm and farmer characteristics, in addition to the defined categories per variable and resulting percentage of farmers per category.

Variable	Question	Answer Format	Category	Farmers (%)
Country	-	-	Italy	16
			Netherlands	45
			Serbia	29
			United Kingdom	11
Arable land	What is the size of your arable land in hectares?	Size in ha	>100	36
			20–100	52
			<20	12
Wheat area	What is the approximate number of hectares of wheat that you cultivate?	Size in ha	>75%	5
			25–75%	66
			<25%	29
Soil type	What is the predominant soil type on which you normally grow wheat?	Multiple choice	Chernozem	19
			Clay	49
			Loam	12
			Loess	8
			Peat	4
			Sand	8
Organic	Do you produce organic wheat?	Yes/No	-	
Main crop	What is the most important crop at your arable farm?	Multiple choice	Maize	19
			Other	17
			Potatoes	36
			Wheat	27
Selling	Do you sell your wheat via a collector/merchant, directly to a feed or food producer or to others?	Multiple choice	Collector	83
			Other	6
			Producer	11
Wheat purpose	Do you grow wheat for human consumption, animal feed or seed production?	Multiple choice	Food	55
			Feed	41
			Seed	5
Benchmark	Do you expect to use this measure against *Fusarium* spp. infection in the coming year? Selection of a *Fusarium* spp. resistant wheat variety Fungicide use around flowering Ploughing after grain harvest Crop rotation: no grains as pre-crop	Yes/No	No	32
			Yes	68
Past infection	How often you think you have had a serious *Fusarium* spp. infection in wheat in the past 5 years?	6-point scale: <never to five times>	No	39
			1	36
			2–5	25
Decision support system-use	Do you use a decision support system to select appropriate measures against *Fusarium* spp. infection?	Yes/No	No	73
			Yes	27
Decision support system-need	Are you in need of a supportive online program that can help you with a choice for an approach to control *Fusarium* spp. infection in wheat?	5-point scale: <Not probable to very probable>	No	8
			Maybe	34
			Yes	58
Gender	What is your gender?	Male/Female	-	
Age	What is your age?	Ten-year age categories	<35	24
			35–44	17
			45–54	30
			55–64	20
			>64	10
Education	What is your highest level of education completed?	Eight educational categories	Primary/Secondary	22
			Vocational	63
			University	16
Risk aversion	Are you willing to take more or less risk regarding *Fusarium* spp. infection and mycotoxin contamination compared to other farmers in your community?	5-point scale: <more to less risk>	Yes	44
			No	47
Risk perception	Do you expect a serious *Fusarium* spp. infection in the coming five years? What consequences will this have?	5-point scale: <never to often>5-point scale: <no consequences to significant consequences>	Low	42
			Medium	45
			High	13
Knowledge	Indicate whether you agree or disagree with the following statements: Harvest debris in the soil forms a risk for *Fusarium* spp. infection You can recognize a *Fusarium* spp. infection by black kernels *Fusarium* species can also be present in maize and barley *Fusarium* species produce mycotoxins like deoxynivalenol Mycotoxins could be harmful to humans	Agree/Disagree/Do not know	Low	11
			Medium	41
			High	48

**Table 2:** Conditional probabilities of farm and farmer characteristics ^a^ of farmers with a decreased (Dec), increased (Inc), or unaltered (Una) intention under the incentives “paid extra”, “no delivery”, “free test”, “insurance”, “contract”. Only results indicating a large numeric difference (>30%) in the conditional probabilities of a characteristic category between farmers who were incentivized (i.e., had an increased intention) or not (i.e., had a decreased intention) are shown. The complete conditional probability tables of each farm and farmer characteristic under each incentive mechanism ^b^ are shown in the Appendix A (Table A2).

		Paid Extra			No Delivery			Free Test			Insurance			Contract		
Variable	Category	Dec (%)	Inc (%)	Una (%)	Dec (%)	Inc (%)	Una (%)	Dec (%)	Inc (%)	Una (%)	Dec (%)	Inc (%)	Una (%)	Dec (%)	Inc (%)	Una (%)
Country	Italy	11	16	3				6	18	6	7	26	7			
	Netherlands	22	56	54				73	42	50	66	22	43			
	Serbia	33	15	33				11	21	33	14	30	36			
	United Kingdom	33	13	9				11	19	11	12	22	14			
Main crop	Maize							0	16	22	11	22	18	17	15	13
	Other							16	18	11	9	22	21	14	18	13
	Potatoes							68	32	47	59	11	39	60	26	45
	Wheat							16	35	19	21	44	21	9	41	29
Education	Primary-Secondary				24	13	17	6	23	14	7	33	22			
	Vocational				76	55	70	89	53	72	84	33	57			
	University				0	31	13	6	25	14	9	33	22			
Soil type	Chernozem	28	8	27							14	26	14			
	Clay	11	60	57							63	29	50			
	Loam	28	10	3							7	15	14			
	Loess	17	10	3							5	15	11			
	Peat	0	2	3							2	0	4			
	Sand	17	10	6							9	15	7			
																
Arable land	Large (>100 ha)	61	26	33												
	Medium (20–100 ha)	17	61	60												
	Small (<20 ha)	22	13	6												
Knowledge	Low										5	11	18			
	Medium										54	15	36			
	High										40	74	46			
Crop purpose	Food													34	64	58
	Feed													63	31	37
	Seed													3	5	6

^a^ For detailed information on the farm and farmer characteristics, see Table 1 and the Methods, Section 4.1.3. ^b^ The conditional probability tables for the incentives “paid less”, “free test”, and “law” are not shown because of the minor differences between the probabilities of farmers with an increased or decreased intention under these incentives; these results are presented in the Appendix A (Table A2).

**Table 3:** Question and answer format of the questionnaire for intention.

Question ^a^
I expect to change my approach to reduce *Fusarium* spp. infection in the coming 5 years.
I plan to change my approach to reduce *Fusarium* spp. infection in the coming 5 years.
I want to change my approach to reduce *Fusarium* spp. infection in the coming 5 years.

^a^ The answer formats were text-only and reflected a 5-point bipolar Likert scale, ranging from strongly disagree to strongly agree.

**Table 4:** Question and answer format of the questionnaire for selected incentives.

Incentive	I Want to Change My Approach to Reduce *Fusarium* spp. infection in the Coming 5 Years if... ^a^
Paid extra	…I get paid extra when my wheat contains low levels of mycotoxins.
Paid less	…I get paid less when my wheat contains too many mycotoxins.
No delivery	…I am not allowed to deliver my wheat because of high mycotoxins levels.
Free advice	…I get free agronomy advice in exchange.
Free test	…I can test my wheat for mycotoxins for free.
Insurance	…I can take out insurance for high mycotoxins levels.
Contract	...that is demanded from the buyer where I can enter a multiyear contract stating a fixed wheat price.
Law	...that is required by law.

^a^ The answer formats were text-only and reflected a 5-point bipolar Likert scale, ranging from strongly disagree to strongly agree.

## Data Availability

The data presented in this study will be openly available in [repository name] at [doi], reference number [reference number] upon publication.

## Appendix A

**Table A1:** Conditional probabilities of farm and farmer characteristics of farmers with a negative, positive, and neutral intention (INT).

		“INT”		
		Negative	Positive	Neutral
Country	Italy	0.18	0.04	0.22
	Netherlands	0.43	0.49	0.59
	Serbia	0.14	0.37	0.04
	United Kingdom	0.25	0.11	0.15
Soil type	Chernozem	0.11	0.25	0.08
	Clay	0.46	0.49	0.62
	Loam	0.21	0.07	0.08
	Loess	0.11	0.05	0.15
	Peat	0.00	0.04	0.00
	Sand	0.11	0.11	0.08
Arable land	Small	0.07	0.12	0.19
	Medium	0.50	0.59	0.44
	Large	0.43	0.28	0.37
Main crop	Wheat	0.39	0.21	0.26
	Maize	0.18	0.19	0.04
	Potatoes	0.25	0.44	0.59
	Other	0.18	0.16	0.11
Wheat area	Small	0.32	0.32	0.22
	Medium	0.64	0.66	0.74
	Large	0.04	0.02	0.04
Crop buyer	Collector	0.85	0.89	0.88
	Producer	0.07	0.09	0.00
	Other	0.07	0.02	0.11
Crop purpose	Food	0.64	0.49	0.48
	Feed	0.32	0.46	0.48
	Seed	0.04	0.05	0.04
Benchmark	No	0.46	0.25	0.45
	Yes	0.54	0.75	0.55
Past infection	No	0.43	0.37	0.52
	One year	0.46	0.39	0.37
	2 to 5 years	0.11	0.25	0.11
DSS use	No	0.61	0.75	0.63
	Yes	0.39	0.25	0.37
DSS need	No	0.04	0.05	0.08
	Maybe	0.43	0.25	0.41
	Yes	0.53	0.70	0.52
Age	Under 35 years	0.14	0.26	0.15
	35 to 44 years	0.14	0.16	0.15
	45 to 54 years	0.32	0.32	0.30
	55 to 64 years	0.28	0.16	0.37
	Over 64 years	0.11	0.11	0.04
Education	Primary/secondary	0.18	0.12	0.26
	Vocational	0.50	0.75	0.59
	University	v0.32	0.12	0.15
Knowledge	Low	0.04	0.12	0.11
	Medium	0.36	0.40	0.44
	High	0.60	0.47	0.44
Risk perception	Low	0.50	0.40	0.41
	Medium	0.39	0.40	0.48
	High	0.11	0.19	0.11
Risk aversion	No	0.36	0.46	0.52
	Yes	0.64	0.54	0.48

**Table A2:** Conditional probabilities of farm and farmer characteristics of farmers with a decreased, increased, or unaltered intention under eight incentives.

		**“Paid Extra”**			**“Paid Less”**		
		**Decreased**	**Increased**	**Unaltered**	**Decreased**	**Increased**	**Unaltered**
Country	Italy	0.11	0.16	0.03	0.13	0.17	0.03
	Netherlands	0.22	0.56	0.54	0.53	0.39	0.61
	Serbia	0.33	0.15	0.33	0.27	0.15	0.31
	United Kingdom	0.33	0.13	0.09	0.07	0.28	0.06
Soil type	Chernozem	0.28	0.08	0.27	0.17	0.13	0.22
	Clay	0.11	0.60	0.57	0.46	0.45	0.63
	Loam	0.28	0.10	0.03	0.13	0.17	0.00
	Loess	0.17	0.10	0.03	0.10	0.13	0.03
	Peat	0.00	0.02	0.03	0.00	0.00	0.06
	Sand	0.17	0.10	0.06	0.13	0.11	0.06
Arable land	Small	0.22	0.13	0.06	0.17	0.15	0.06
	Medium	0.17	0.61	0.60	0.50	0.41	0.72
	Large	0.61	0.26	0.33	0.33	0.43	0.22
Main crop	Wheat	0.22	0.13	0.15	0.20	0.11	0.17
	Maize	0.06	0.03	0.00	0.04	0.02	0.03
	Potatoes	0.22	0.26	0.39	0.23	0.26	0.39
	Other	0.72	0.70	0.60	0.73	0.71	0.58
Wheat area	Small	0.44	0.26	0.18	0.27	0.37	0.14
	Medium	0.28	0.47	0.42	0.53	0.35	0.44
	Large	0.06	0.13	0.24	0.00	0.17	0.25
Crop buyer	Collector	0.82	0.90	0.87	0.96	0.82	0.88
	Producer	0.17	0.05	0.03	0.04	0.11	0.03
	Other	0.01	0.05	0.09	0.00	0.07	0.09
Crop purpose	Food	0.66	0.52	0.45	0.47	0.59	0.50
	Feed	0.33	0.41	0.51	0.50	0.37	0.44
	Seed	0.01	0.07	0.03	0.04	0.05	0.06
Benchmark	No	0.23	0.48	0.19	0.30	0.46	0.25
	Yes	0.77	0.52	0.82	0.70	0.54	0.75
Past infection	No	0.50	0.39	0.42	0.43	0.43	0.39
	One year	0.39	0.39	0.42	0.43	0.39	0.39
	2 to 5 years	0.12	0.21	0.15	0.14	0.18	0.22
DSS use	No	0.72	0.65	0.73	0.73	0.56	0.80
	Yes	0.28	0.35	0.28	0.27	0.44	0.20
DSS need	No	0.06	0.07	0.03	0.10	0.05	0.03
	Maybe	0.50	0.38	0.15	0.40	0.39	0.20
	Yes	0.44	0.56	0.81	0.50	0.56	0.77
Age	Under 35 years	0.33	0.15	0.24	0.23	0.15	0.25
	35 to 44 years	0.17	0.13	0.18	0.20	0.13	0.14
	45 to 54 years	0.22	0.33	0.33	0.27	0.37	0.28
	55 to 64 years	0.28	0.26	0.18	0.17	0.28	0.25
	Over 64 years	0.00	0.13	0.06	0.13	0.07	0.08
Education	Primary/secondary	0.17	0.21	0.09	0.17	0.22	0.11
	Vocational	0.55	0.59	0.81	0.70	0.50	0.80
	University	0.28	0.20	0.09	0.14	0.28	0.09
Knowledge	Low	0.01	0.12	0.12	0.04	0.07	0.20
	Medium	0.39	0.36	0.48	0.33	0.46	0.39
	High	0.61	0.52	0.39	0.63	0.48	0.42
Risk perception	Low	0.50	0.41	0.42	0.40	0.52	0.33
	Medium	0.33	0.49	0.33	0.50	0.37	0.42
	High	0.17	0.10	0.24	0.10	0.11	0.25
Risk aversion	No	0.28	0.52	0.40	0.34	0.50	0.47
	Yes	0.72	0.48	0.61	0.66	0.50	0.53
INT	Negative	0.28	0.33	0.09	0.20	0.39	0.11
	Neutral	0.12	0.33	0.15	0.10	0.37	0.20
	Positive	0.61	0.34	0.75	0.70	0.24	0.69
INCp	Negative	0.50	0.02	0.09	0.66	0.00	0.09
	Neutral	0.44	0.02	0.18	0.30	0.11	0.25
	Positive	0.06	0.96	0.72	0.04	0.89	0.66
				**“No Delivery”**		**“Free Advice”**	
		**Decreased**	**Increased**	**Unaltered**	**Decreased**	**Increased**	**Unaltered**
Country	Italy	0.03	0.15	0.17	0.08	0.15	0.12
	Netherlands	0.64	0.44	0.41	0.58	0.52	0.38
	Serbia	0.26	0.19	0.29	0.11	0.25	0.35
	United Kingdom	0.06	0.22	0.13	0.24	0.08	0.15
Soil type	Chernozem	0.18	0.19	0.13	0.16	0.15	0.21
	Clay	0.58	0.48	0.50	0.50	0.60	0.44
	Loam	0.03	0.15	0.13	0.11	0.13	0.09
	Loess	0.09	0.09	0.08	0.05	0.10	0.12
	Peat	0.06	0.00	0.00	0.03	0.03	0.00
	Sand	0.06	0.09	0.17	0.16	0.00	0.15
Arable land	Small	0.09	0.17	0.09	0.03	0.18	0.18
	Medium	0.70	0.43	0.54	0.52	0.60	0.47
	Large	0.21	0.41	0.37	0.45	0.23	0.35
Main crop	Wheat	0.21	0.31	0.25	0.26	0.23	0.32
	Maize	0.15	0.15	0.17	0.05	0.20	0.21
	Potatoes	0.61	0.39	0.25	0.52	0.40	0.35
	Other	0.03	0.15	0.33	0.16	0.18	0.12
Wheat area	Small	0.32	0.22	0.42	0.34	0.25	0.29
	Medium	0.64	0.76	0.54	0.63	0.70	0.70
	Large	0.03	0.02	0.05	0.03	0.05	0.00
Crop buyer	Collector	0.91	0.90	0.79	0.86	0.92	0.85
	Producer	0.06	0.06	0.09	0.11	0.05	0.03
	Other	0.03	0.04	0.13	0.03	0.03	0.12
Crop purpose	Food	0.50	0.55	0.50	0.42	0.55	0.61
	Feed	0.47	0.39	0.46	0.52	0.40	0.35
	Seed	0.03	0.06	0.05	0.06	0.05	0.03
Benchmark	No	0.30	0.37	0.38	0.27	0.43	0.35
	Yes	0.70	0.63	0.62	0.73	0.57	0.65
Past infection	No	0.35	0.44	0.46	0.37	0.37	0.53
	One year	0.44	0.41	0.33	0.45	0.40	0.35
	2 to 5 years	0.21	0.15	0.21	0.19	0.23	0.12
DSS use	No	0.79	0.63	0.66	0.66	0.62	0.79
	Yes	0.21	0.37	0.34	0.34	0.38	0.21
DSS need	No	0.09	0.06	0.00	0.03	0.03	0.12
	Maybe	0.27	0.39	0.29	0.47	0.28	0.24
	Yes	0.64	0.55	0.70	0.50	0.70	0.64
Age	Under 35 years	0.12	0.22	0.29	0.18	0.13	0.32
	35 to 44 years	0.15	0.13	0.21	0.24	0.08	0.15
	45 to 54 years	0.32	0.30	0.33	0.29	0.32	0.32
	55 to 64 years	0.23	0.28	0.17	0.26	0.30	0.15
	Over 64 years	0.18	0.07	0.00	0.03	0.18	0.06
Education	Primary/secondary	0.24	0.13	0.17	0.08	0.25	0.18
	Vocational	0.76	0.55	0.70	0.76	0.57	0.61
	University	0.00	0.31	0.13	0.16	0.18	0.21
Knowledge	Low	0.09	0.08	0.17	0.08	0.10	0.12
	Medium	0.50	0.35	0.37	0.47	0.42	0.29
	High	0.41	0.57	0.46	0.45	0.47	0.59
Risk perception	Low	0.32	0.43	0.58	0.42	0.33	0.56
	Medium	0.47	0.43	0.33	0.39	0.57	0.27
	High	0.21	0.15	0.09	0.19	0.10	0.18
Risk aversion	No	0.44	0.48	0.38	0.42	0.48	0.44
	Yes	0.56	0.52	0.62	0.58	0.52	0.56
INT	Negative	0.06	0.41	0.17	0.19	0.35	0.21
	Neutral	0.24	0.28	0.17	0.19	0.25	0.29
	Positive	0.70	0.31	0.66	0.63	0.40	0.50
INCp	Negative	0.79	0.00	0.13	0.58	0.03	0.15
	Neutral	0.18	0.02	0.25	0.37	0.15	0.41
	Positive	0.03	0.98	0.62	0.06	0.82	0.44
		**“Free Test”**			**“Insurance”**		
		**Decreased**	**Increased**	**Unaltered**	**Decreased**	**Increased**	**Unaltered**
Country	Italy	0.06	0.18	0.06	0.07	0.26	0.07
	Netherlands	0.73	0.42	0.50	0.66	0.22	0.43
	Serbia	0.11	0.21	0.33	0.14	0.30	0.36
	United Kingdom	0.11	0.19	0.11	0.12	0.22	0.14
Soil type	Chernozem	0.11	0.14	0.25	0.14	0.26	0.14
	Clay	0.68	0.49	0.47	0.63	0.29	0.50
	Loam	0.05	0.16	0.06	0.07	0.15	0.14
	Loess	0.11	0.09	0.08	0.05	0.15	0.11
	Peat	0.05	0.02	0.00	0.02	0.00	0.04
	Sand	0.00	0.11	0.14	0.09	0.15	0.07
Arable land	Small	0.01	0.18	0.11	0.05	0.30	0.11
	Medium	0.52	0.53	0.55	0.59	0.37	0.57
	Large	0.47	0.30	0.33	0.35	0.33	0.32
Main crop	Wheat	0.16	0.35	0.19	0.21	0.44	0.21
	Maize	0.00	0.16	0.22	0.11	0.22	0.18
	Potatoes	0.68	0.32	0.47	0.59	0.11	0.39
	Other	0.16	0.18	0.11	0.09	0.22	0.21
Wheat area	Small	0.26	0.21	0.44	0.30	0.22	0.36
	Medium	0.73	0.73	0.55	0.68	0.70	0.64
	Large	0.01	0.05	0.00	0.02	0.08	0.00
Crop buyer	Collector	0.89	0.89	0.86	0.93	0.85	0.82
	Producer	0.06	0.07	0.06	0.05	0.08	0.07
	Other	0.06	0.04	0.09	0.02	0.08	0.11
Crop purpose	Food	0.37	0.59	0.50	0.46	0.66	0.53
	Feed	0.57	0.37	0.44	0.51	0.33	0.36
	Seed	0.06	0.04	0.06	0.04	0.00	0.11
Benchmark	No	0.22	0.39	0.36	0.26	0.45	0.43
	Yes	0.78	0.61	0.64	0.74	0.55	0.57
Past infection	No	0.26	0.40	0.53	0.39	0.37	0.53
	One year	0.47	0.40	0.36	0.39	0.44	0.39
	2 to 5 years	0.26	0.19	0.11	0.23	0.19	0.07
DSS use	No	0.68	0.60	0.83	0.75	0.48	0.75
	Yes	0.32	0.40	0.17	0.25	0.52	0.25
DSS need	No	0.06	0.07	0.03	0.04	0.11	0.04
	Maybe	0.42	0.35	0.25	0.30	0.44	0.29
	Yes	0.52	0.58	0.72	0.66	0.44	0.67
Age	Under 35 years	0.06	0.19	0.30	0.18	0.19	0.28
	35 to 44 years	0.16	0.18	0.11	0.21	0.11	0.07
	45 to 54 years	0.42	0.32	0.25	0.26	0.40	0.32
	55 to 64 years	0.31	0.21	0.25	0.26	0.19	0.25
	Over 64 years	0.06	0.11	0.08	0.09	0.11	0.07
Education	Primary/secondary	0.06	0.23	0.14	0.07	0.33	0.22
	Vocational	0.89	0.53	0.72	0.84	0.33	0.57
	University	0.06	0.25	0.14	0.09	0.33	0.22
Knowledge	Low	0.06	0.12	0.09	0.05	0.11	0.18
	Medium	0.52	0.35	0.42	0.54	0.15	0.36
	High	0.42	0.53	0.50	0.40	0.74	0.46
Risk perception	Low	0.52	0.40	0.42	0.40	0.44	0.46
	Medium	0.26	0.47	0.42	0.44	0.37	0.43
	High	0.21	0.12	0.17	0.16	0.19	0.11
Risk aversion	No	0.47	0.44	0.44	0.42	0.41	0.54
	Yes	0.53	0.56	0.56	0.58	0.59	0.46
INT	Negative	0.16	0.37	0.11	0.16	0.48	0.22
	Neutral	0.16	0.25	0.28	0.21	0.19	0.36
	Positive	0.68	0.39	0.61	0.63	0.33	0.43
INCp	Negative	0.47	0.02	0.09	0.70	0.08	0.18
	Neutral	0.42	0.11	0.39	0.28	0.26	0.46
	Positive	0.11	0.87	0.53	0.02	0.66	0.36
		**“Contract”**			**“Law”**		
		**Decreased**	**Increased**	**Unaltered**	**Decreased**	**Increased**	**Unaltered**
Country	Italy	0.06	0.18	0.11	0.03	0.17	0.13
	Netherlands	0.65	0.38	0.47	0.64	0.37	0.53
	Serbia	0.14	0.26	0.29	0.18	0.23	0.30
	United Kingdom	0.14	0.18	0.13	0.15	0.23	0.04
Soil type	Chernozem	0.14	0.18	0.18	0.15	0.23	0.10
	Clay	0.62	0.43	0.50	0.56	0.44	0.60
	Loam	0.09	0.18	0.05	0.12	0.15	0.03
	Loess	0.09	0.08	0.11	0.09	0.06	0.13
	Peat	0.00	0.03	0.03	0.03	0.00	0.03
	Sand	0.06	0.10	0.13	0.06	0.13	0.10
Arable land	Small	0.03	0.21	0.13	0.03	0.17	0.17
	Medium	0.68	0.38	0.55	0.64	0.46	0.53
	Large	0.29	0.41	0.32	0.32	0.37	0.30
Main crop	Wheat	0.09	0.41	0.29	0.18	0.35	0.23
	Maize	0.17	0.15	0.13	0.12	0.17	0.17
	Potatoes	0.60	0.26	0.45	0.58	0.31	0.43
	Other	0.14	0.18	0.13	0.12	0.17	0.17
Wheat area	Small	0.31	0.28	0.29	0.32	0.29	0.27
	Medium	0.68	0.66	0.68	0.64	0.69	0.70
	Large	0.00	0.05	0.03	0.03	0.02	0.04
Crop buyer	Collector	0.94	0.89	0.81	0.88	0.91	0.83
	Producer	0.06	0.05	0.08	0.12	0.04	0.04
	Other	0.00	0.05	0.11	0.00	0.04	0.14
Crop purpose	Food	0.34	0.64	0.58	0.47	0.58	0.50
	Feed	0.63	0.31	0.37	0.53	0.35	0.43
	Seed	0.03	0.05	0.06	0.00	0.06	0.07
Benchmark	No	0.29	0.44	0.32	0.24	0.46	0.30
	Yes	0.71	0.56	0.68	0.76	0.54	0.70
Past infection	No	0.31	0.38	0.55	0.38	0.44	0.43
	One year	0.46	0.46	0.29	0.47	0.37	0.37
	2 to 5 years	0.23	0.16	0.16	0.15	0.19	0.20
DSS use	No	0.68	0.59	0.79	0.62	0.64	0.83
	Yes	0.32	0.41	0.21	0.38	0.36	0.17
DSS need	No	0.03	0.03	0.11	0.03	0.06	0.07
	Maybe	0.40	0.41	0.19	0.27	0.46	0.20
	Yes	0.57	0.56	0.71	0.70	0.48	0.73
Age	under 35	0.14	0.26	0.21	0.06	0.29	0.23
	35 to 44	0.23	0.10	0.13	0.21	0.13	0.13
	45 to 54	0.26	0.36	0.31	0.35	0.27	0.33
	55 to 64	0.28	0.23	0.21	0.29	0.23	0.20
	over 64	0.09	0.05	0.13	0.09	0.08	0.10
Education	Primary/secondary	0.12	0.23	0.16	0.24	0.15	0.14
	Vocational	0.74	0.51	0.71	0.64	0.54	0.83
	University	0.14	0.26	0.13	0.12	0.31	0.04
Knowledge	Low	0.09	0.05	0.16	0.06	0.09	0.17
	Medium	0.40	0.44	0.37	0.50	0.40	0.30
	High	0.51	0.51	0.47	0.44	0.52	0.53
Risk perception	Low	0.43	0.51	0.34	0.32	0.48	0.47
	Medium	0.40	0.33	0.52	0.50	0.40	0.37
	High	0.17	0.16	0.13	0.18	0.13	0.17
Risk aversion	No	0.43	0.54	0.37	0.44	0.46	0.43
	Yes	0.57	0.46	0.63	0.56	0.54	0.57
INT	Negative	0.17	0.46	0.11	0.09	0.42	0.17
	Neutral	0.26	0.21	0.26	0.21	0.29	0.20
	Positive	0.57	0.33	0.63	0.70	0.29	0.63
INCp	Negative	0.71	0.08	0.08	0.67	0.02	0.10
	Neutral	0.26	0.10	0.42	0.24	0.11	0.37
	Positive	0.03	0.82	0.50	0.09	0.87	0.53

**Table A3:** Specific conditional probabilities of farm and farmer characteristics of farmers with a decreased (Dec) or increased (Inc) intention under the most promising incentive, “paid extra”, for farmers per country from Italy (IT), the Netherlands (NL), Serbia (RS), and the United Kingdom (UK) and the combination of the four countries (Eur). The bold values represent the three selected farm and farmer characteristics to run in the Bayesian Network models in scenario 2.

		IT		NL		RS		UK		Eur	
		Dec (%)	Inc (%)	Dec (%)	Inc (%)	Dec (%)	Inc (%)	Dec (%)	Inc (%)	Dec (%)	Inc (%)
Arable land	Small			1	0					22	13
	Medium			1	82					17	61
	Large			**99**	18					**61**	26
											
Main crop	Wheat	**97**	50								
	Maize	1	30								
	Potatoes	1	0								
	Other	1	20								
											
Wheat area	Small	1	10			33	66				
	Medium	49	80			**66**	33				
	Large	**49**	10			0	0				
											
Crop purpose	Food							**99**	62		
	Feed							0	37		
	Seed							0	0		
											
Past infection	No	**78**	37								
	One year	21	45								
	2 to 5 years	1	17								
											
Benchmark	No							19	48	23	48
	Yes							**81**	52	**77**	52
											
Age	Under 35 years			0	9			0	13		
	35 to 44 years			25	18			17	13		
	45 to 54 years			**49**	18			17	50		
	55 to 64 years			25	38			**66**	13		
	Over 64 years			0	18			0	13		
											
Education	Primary/secondary					17	44				
	Vocational					**66**	33				
	University					17	22				
											
Risk aversion	No			10	55					28	52
	Yes			**90**	45					**72**	48
											
Risk perception	Low					**67**	36				
	Medium					26	50				
	High					7	14				
